# The FANCD2–FANCI complex is recruited to DNA interstrand crosslinks before monoubiquitination of FANCD2

**DOI:** 10.1038/ncomms12124

**Published:** 2016-07-13

**Authors:** Chih-Chao Liang, Zhuolun Li, David Lopez-Martinez, William V. Nicholson, Catherine Vénien-Bryan, Martin A. Cohn

**Affiliations:** 1 Department of Biochemistry, University of Oxford, Oxford OX1 3QU, UK; 2 Institute of Mineralogy, Materials Physics and Cosmochemistry, UMR 7590, UPMC, CNRS, IRD, MNHN, Paris F-75005, France

## Abstract

The Fanconi anaemia (FA) pathway is important for the repair of DNA interstrand crosslinks (ICL). The FANCD2–FANCI complex is central to the pathway, and localizes to ICLs dependent on its monoubiquitination. It has remained elusive whether the complex is recruited before or after the critical monoubiquitination. Here, we report the first structural insight into the human FANCD2–FANCI complex by obtaining the cryo-EM structure. The complex contains an inner cavity, large enough to accommodate a double-stranded DNA helix, as well as a protruding Tower domain. Disease-causing mutations in the Tower domain are observed in several FA patients. Our work reveals that recruitment of the complex to a stalled replication fork serves as the trigger for the activating monoubiquitination event. Taken together, our results uncover the mechanism of how the FANCD2–FANCI complex activates the FA pathway, and explains the underlying molecular defect in FA patients with mutations in the Tower domain.

## Abstract (web summary)

FANCD2 and FANCI are essential components of the Fanconi anaemia DNA damage repair pathway. Here the authors present the cryo-EM structure of the FANCD2-FANCI complex, providing insight into how the complex is recruited to stalled replication forks.

## Graphical abstract

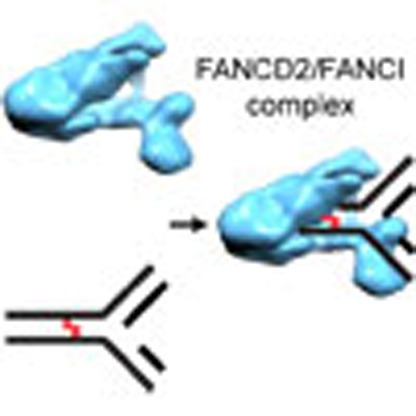

Fanconi anaemia (FA) is a genetic disorder characterized by developmental defects, cancer susceptibility and a severe cellular sensitivity to agents inducing toxic interstrand crosslinks (ICLs) formed between the two strands of the Watson–Crick DNA helix. A total of 19 FA proteins function together in a complex pathway leading to the repair of an ICL, primarily in the S-phase of the cell cycle^1^. Central to the pathway is the FANCD2–FANCI protein complex, recruitment of which to the ICL is critical for repair. It is known that monoubiquitination of FANCD2 is indispensable to a functional pathway^2^, and several studies demonstrate how monoubiquitinated FANCD2 facilitates recruitment of the FAN1 (refs 3, 4, 5), and XPF-ERCC1 (refs 6, 7) nucleases to the ICL, providing a molecular explanation for why monoubiquitination of FANCD2 is critical to the pathway. The underlying mechanism for recruitment of the FANCD2–FANCI complex itself to the ICL is currently unclear.

Whereas the structure of the mouse homologues has been reported^8^, to date there is no structural information on the human FANCD2–FANCI complex. Here, we present a cryo-EM structure of the full-length human FANCD2–FANCI complex, demonstrating the existence of a novel Tower domain. The Tower domain is required for recruitment of the complex to ICLs *in vitro* and *in vivo*. We also demonstrate, for the fist time, a direct interaction between the FANCD2–FANCI complex and a DNA structure resembling a replication fork stalled at an ICL. Finally, we present data firmly establishing that the FANCD2–FANCI complex needs to be bound to DNA, to be efficiently monoubiquitinated. Several FA disease-causing mutations lie within the Tower domain, thus we have uncovered the molecular basis for the disease in these patients.

## Results

### Cryo-EM structure of the full-length FANCD2–FANCI complex

**Figure 1:**
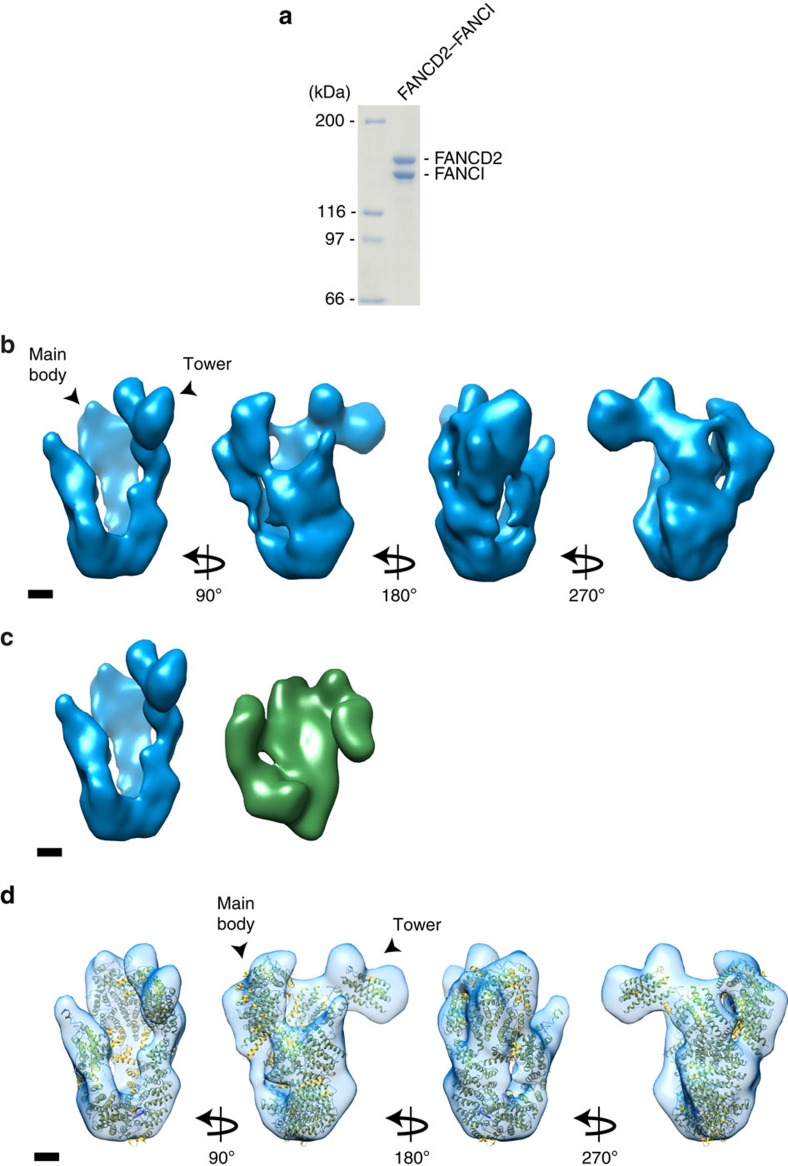
Cryo-EM structure of the FANCD2–FANCI complex. (**a**) Coomassie blue stain of recombinant full-length FANCD2–FANCI heterodimer purified from Sf9 cells. (**b**) Cryo-EM density map of full-length FANCD2–FANCI complex in different orientations. Scale bar, 20 Å. (**c**) Comparison of full-length FANCD2–FANCI Cryo-EM structure (left) with the mFANCD2–FANCI crystal structure (PDB: 3S4W) (right). The mFANCD2–FANCI crystal structure was filtered to the same resolution as the 3D Cryo-EM model. Scale bar, 20 Å. (**d**) Different orientations of Cryo-EM density map of full-length FANCD2–FANCI complex docked with the mFANCD2–FANCI crystal structure (PDB: 3S4W). Scale bar, 20 Å.

To gain further insight into the mechanism of FANCD2–FANCI function during ICL repair, we decided to obtain structural insight of the full-length human complex by electron microscopy (EM). We co-expressed Flag-HA-tagged human FANCD2 and His-tagged human FANCI in Sf9 cells and purified the heterodimeric complex to homogeneity (Fig. 1a). The intensities of FANCD2 and FANCI in the Coomassie-stained gel appear identical. The highly pure complex was then subjected to EM analysis using negative stain. Representative micrographs of the grids demonstrated homogeneity of the sample (Supplementary Fig. 1a,b). In all, 4,082 pairs of particles were used to calculate a three-dimensional (3D) model using the random conical tilt method (RCT)^9^.

**Figure 2:**
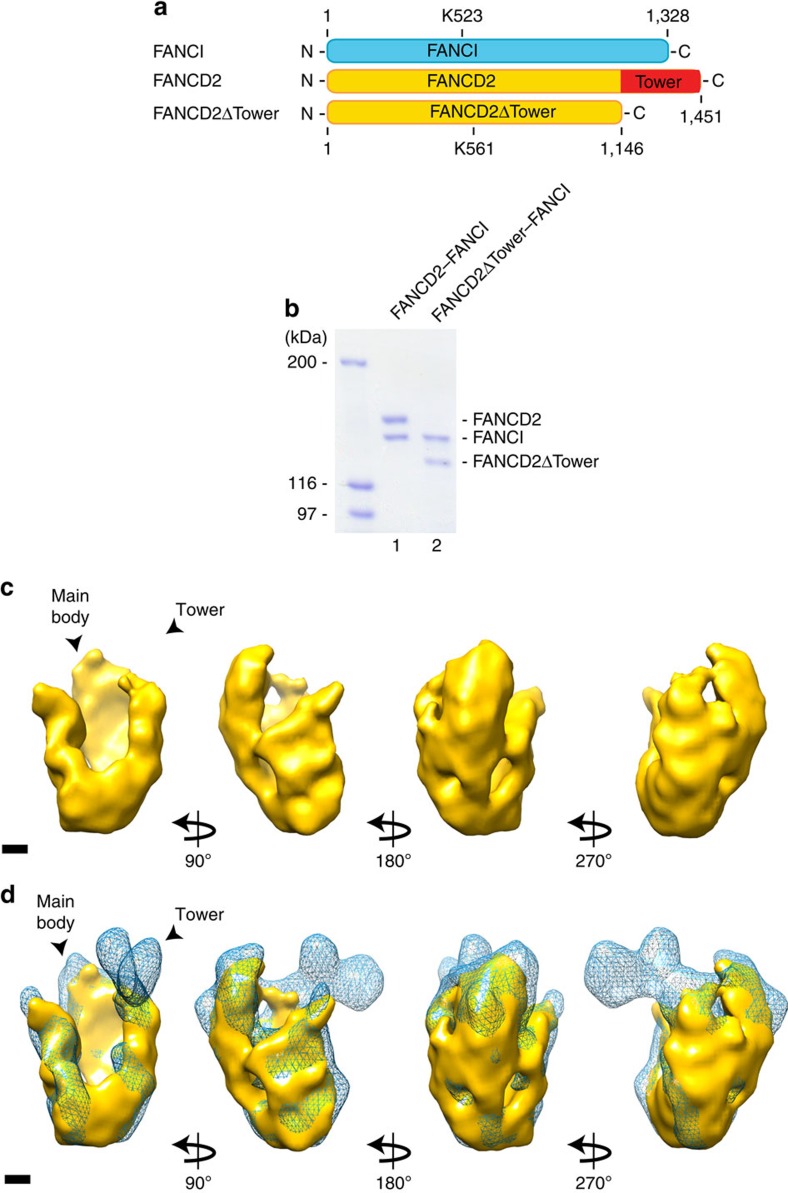
Determination of the Tower domain of FANCD2. (**a**) Schematic of FANCI and FANCD2 indicating the Tower domain. (**b**) Coomassie blue stain of recombinant full-length FANCD2–FANCI and FANCD2ΔTower–FANCI heterodimer purified from Sf9 cells. (**c**) Different orientations of Cryo-EM density map of the FANCD2ΔTower–FANCI complex. Scale bar, 20 Å. (**d**) Different orientations of Cryo-EM density map of full-length FANCD2–FANCI complex (in blue mesh) superimposed with the FANCD2ΔTower–FANCI complex (in gold). Scale bar, 20 Å.

We then subjected the FANCD2–FANCI complex to cryo-EM to refine this preliminary 3D model. A micrograph shows homogeneity of the sample (Supplementary Fig. 2a), from which we obtained a structure with a resolution of 22 Å, allowing the determination of further details of the structure. The 3D-reconstruction revealed a 90 Å wide and 160 Å long structure with a hollow central part. We also observed a characteristic 60 Å protruding domain, which we termed as the Tower domain (Fig. 1b). We noticed that the end of the Tower domain adapts a fork-like structure. When comparing our determined structure with the crystal structure of the mouse FANCD2–FANCI complex^8^, we noticed a striking difference. While the position of the Tower domain is vertical in our structure, it adapts a horizontal position in the crystal structure (Fig. 1c). When we dock the crystal structure onto the cryo-EM structure, allowing flexibility of each of the eight solenoids in the crystal structure relative to each other, we observe good agreement between the two structures (Fig. 1d). The difference in the position of the Tower domain in the two structures could be a result of the two different methods used to obtain the structures. The crystal structure is based on a version of FANCD2 containing three relatively large deletions, whereas the EM structure is based on the full-length protein, which might also contribute to the observed differences. The position of the Tower domain suggested that it is composed of the C-terminus of FANCD2. To test this directly, we introduced a C-terminal deletion in FANCD2 containing the last 305 amino acids (Fig. 2a), and purified the complex of this protein (FANCD2ΔTower) and FANCI (Fig. 2b). The highly purified FANCD2ΔTower-FANCI heterodimer was subjected to cryo-EM, resulting in a structure at 20 Å resolution clearly missing the Tower domain, but otherwise similar to the full-length structure (Fig. 2c,d). These data are further confirmed by docking the mouse crystal structure, where we have removed the C-terminal 305 amino acids onto the cryo-EM structure of the FANCD2ΔTower–FANCI complex (Supplementary Fig. 2b).

### The Tower domain is important for the FANCD2–FANCI complex

**Figure 3:**
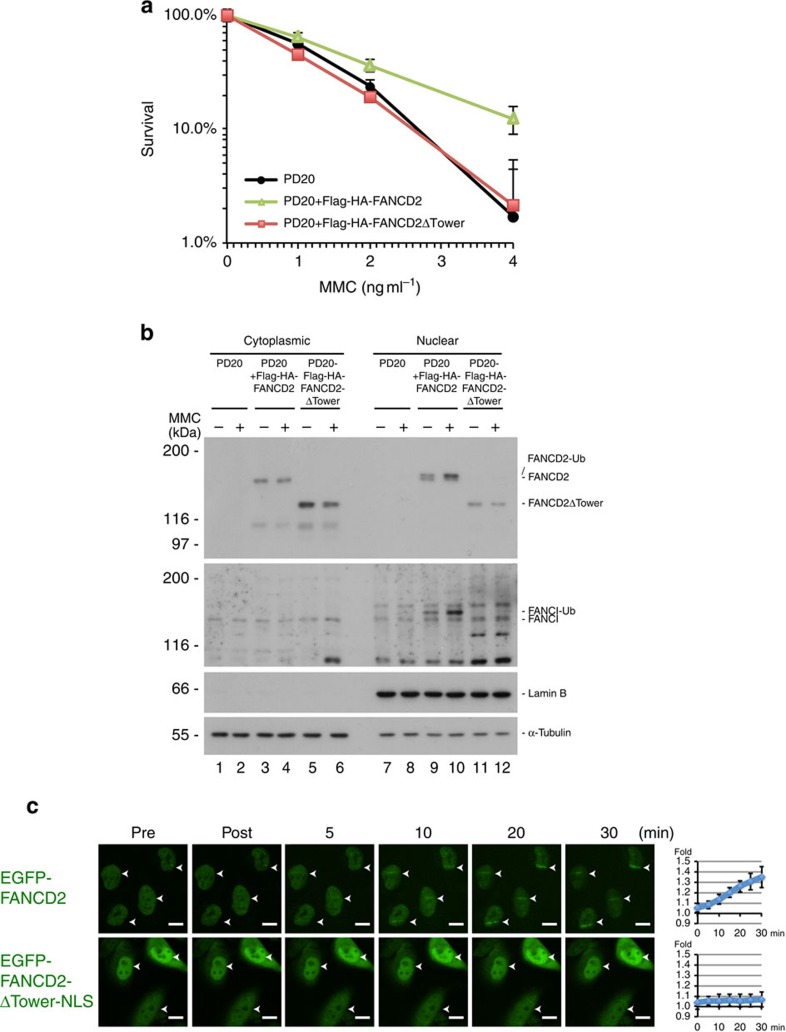
The Tower domain is critical for FANCD2 function and the FA pathway. (**a**) Clonogenic survival assay of PD20 cells complemented with Flag-HA-FANCD2 or Flag-HA-FANCD2ΔTower. The experiment was done in triplicate. (**b**) FANCD2 monoubiquitination response in PD20 cells complemented with wild-type Flag-HA-FANCD2 or Flag-HA-FANCD2ΔTower after 160 ng ml^−1^ MMC overnight treatment. (**c**) HeLa shFANCD2 cells expressing EGFP–FANCD2 or EGFP–FANCD2ΔTower-NLS were pre-treated with TMP, and micro-irradiated at the indicated area (white arrows). Wild-type FANCD2 was recruited to TMP-induced ICLs sites, while FANCD2ΔTower-NLS was not. Representative fields shown. Quantifications of EGFP–FANCD2 (six cells quantified) or EGFP–FANCD2ΔTower-NLS (six cells quantified) at the irradiated sites are shown at the right. Scale bars, 20 μm. Error bars in **a**–**c** show s.d.

Given the striking position of the Tower domain, we decided to determine whether this domain is functionally important to the FANCD2–FANCI complex, and to the FA pathway. We stably expressed exogenous full-length FANCD2 or FANCD2ΔTower in patient-derived FANCD2-deficient PD20 cells (Supplementary Fig. 3a). The ability of these cell lines to survive under increasing concentrations of the ICL-inducing drug mitomycin C (MMC) was then assessed. We found that non-complemented cells were very sensitive to MMC, and that expression of the full-length protein restored resistance (Fig. 3a). On the other hand, expression of the FANCD2ΔTower protein did not complement the cells, demonstrating the functional importance of the Tower domain (Fig. 3a).

Monoubiquitination of FANCD2 is necessary for its ICL DNA-repair function. We speculated that the Tower domain might affect the ability of FANCD2 to be monoubiquitinated, and that this could be part of the mechanism underlying its function in the pathway. We assessed the ability of the FANCD2 and FANCD2ΔTower proteins expressed in PD20 cells to be monoubiquitinated. As expected, full-length FANCD2 was strongly ubiquitinated in response to MMC (Fig. 3b, lane 10). In contrast, the FANCD2ΔTower protein was not ubiquitinated at all (Fig. 3b, lane 12). Also, we observed a nice restoration of FANCI ubiquitination when full-length FANCD2 was expressed, but no ubiquitination of FANCI when the FANCD2ΔTower protein was expressed. The absence of ubiquitination of FANCD2 could be due to lack of interaction with its E3 ubiquitin ligase complex (core complex). However, both full-length and the Tower-deletion protein interact equally well with the core complex (Supplementary Fig. 3b). Since the Tower domain is important for the function of FANCD2, and also for its monoubiquitination, we next assessed whether the Tower domain is important for the recruitment of FANCD2 to ICLs *in vivo*. To this end we utilized a live-cell imaging system that we have previously described^10^. We reduced the cellular levels of endogenous FANCD2 in HeLa cells by shRNA and then stably expressed exogenous shRNA-resistant EGFP-tagged either full-length FANCD2 or FANCD2ΔTower (Supplementary Fig. 3c), and confirmed the established cell lines by clonogenic survival assays (Supplementary Fig. 3d). Full-length FANCD2 was recruited to ICLs as expected, forming clear stripes 10 min after the introduction of the ICLs (Fig. 3c). Strikingly, deletion of the Tower domain completely abolished the recruitment (Fig. 3c).

### The Tower domain is needed for recruitment of FANCD2 to ICLs

**Figure 4:**
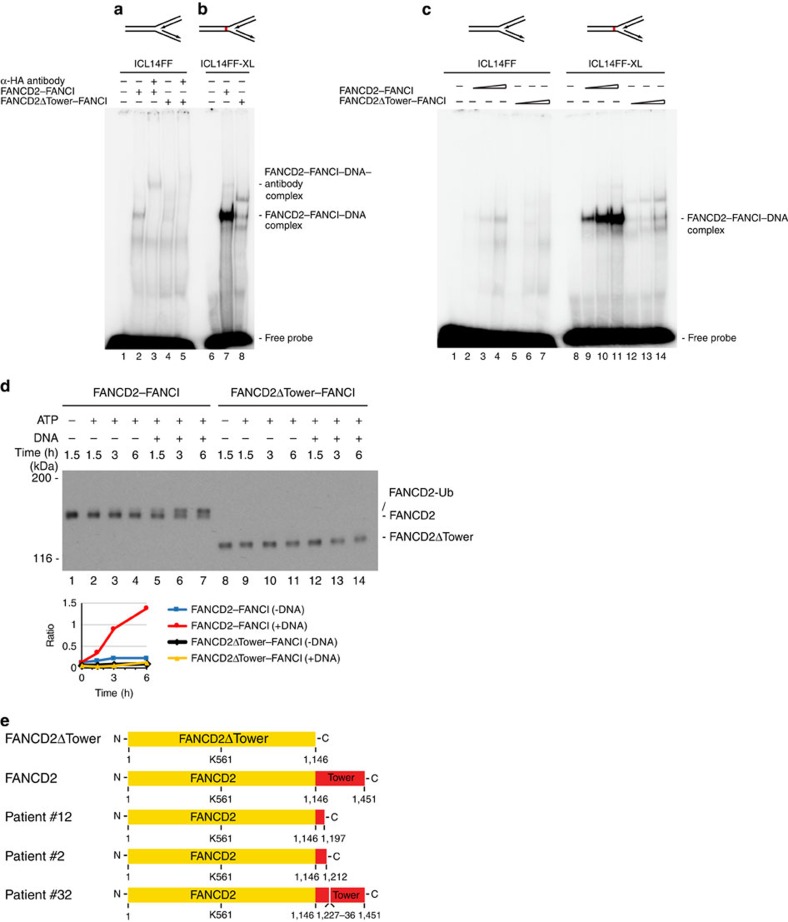
The Tower domain controls binding to DNA and is critical for the monoubiquitination of FANCD2 after binding to DNA. (**a**) EMSA showing full-length FANCD2–FANCI complex forming specific protein–DNA complexes with replication fork substrate ICL14FF, while the FANCD2ΔTower–FANCI complex only forms weak complexes. (**b**) EMSA showing full-length FANCD2–FANCI complex forming stronger specific protein–DNA complexes with crosslinked replication fork substrate ICL14FF-XL, while the FANCD2ΔTower–FANCI complex only forms weak complexes. (**c**) Titration of protein concentration of full-length FANCD2–FANCI complex and FANCD2ΔTower–FANCI complex in EMSA with replication fork substrate ICL14FF (left) and crosslinked replication fork substrate ICL14FF-XL (right). (**d**) *In vitro* ubiquitination assay of full-length FANCD2–FANCI complex and FANCD2ΔTower–FANCI complex. Quantification shows the ratio of monoubiquitinated FANCD2 or FANCD2ΔTower versus unmodified FANCD2 and FANCD2ΔTower at the indicated time points. Error bars show s.d. (**e**) Schematic of patient-derived mutations in the Tower domain that led to deletion of parts of the domain.

Since the Tower domain is functionally important, due to its requirement for monoubiquitination of FANCD2, and its role in the recruitment of FANCD2 to ICLs *in vivo*, we speculated that this domain might be involved in the interaction of the FANCD2–FANCI complex with DNA. To test this directly, we assessed the ability of the FANCD2–FANCI and FANCD2ΔTower–FANCI complexes to interact with DNA. It is presumed that the FANCD2–FANCI complex is recruited to replication forks stalled at an ICL. Therefore, we synthesized a DNA molecule mimicking a replication fork and evaluated the abilities of the two complexes to interact with it. FANCD2–FANCI formed a specific complex with the DNA, which could be super-shifted using specific antibodies (Fig. 4a, lanes 2–3). On the other hand, FANCD2ΔTower–FANCI formed a much weaker complex with the DNA, demonstrating the importance of the Tower domain in the protein–DNA interaction (Fig. 4a, lanes 4–5). To gain further insight into the mechanism of the interaction, we next synthesized a similar DNA molecule, but now containing an ICL in the fork, more realistically representing a replication fork stalled at an ICL. Using this molecule, we again observed near abrogation of DNA binding when the Tower domain was deleted (Fig. 4b). Interestingly, we found that FANCD2–FANCI interacted significantly stronger with the fork containing an ICL, than with the non-crosslinked counterpart. Titrating the amount of protein in the binding reaction confirmed the observed preference (Fig. 4c).

### FANCD2–FANCI is recruited to DNA before ubiquitination

Our data demonstrate a direct interaction between the FANCD2–FANCI complex and a replication fork DNA structure containing an ICL, and that the interaction is dependent on the Tower domain in FANCD2. We also know that the Tower domain is required for monoubiquitination of FANCD2. Therefore, we hypothesized that perhaps the reason the FANCD2ΔTower is not ubiquitinated is that the protein is not recruited to DNA, and therefore not ubiquitinated. If that were the case, it could mean that FANCD2–FANCI has to be bound to DNA to be ubiquitinated. To test this hypothesis, we reconstituted the monoubiquitination reaction *in vitro*, using recombinant proteins (Supplementary Fig. 4a). We then first assessed the monoubiquitination of FANCD2 in the absence of DNA, and observed only very weak monoubiquitination (Fig. 4d, lanes 2–4). However, when we added DNA to the reaction, a robust monoubiquitination was observed (Fig. 4d, lanes 5–7), consistent with a previous report^11^. The increase in specific monoubiquitination was not due to increased general E3 ligase activity in the reaction (Supplementary Fig. 4b). Interestingly, we observed only modest monoubiquitination of FANCI, in good agreement with previous reports (Supplementary Fig. 4c)^11^,^12^. On the other hand, no monoubiquitination was observed when we subjected the FANCD2ΔTower–FANCI complex to the same assay (Fig. 4d, lanes 12–14). Taken together, this suggests that the FANCD2–FANCI complex must be bound to DNA to be activated by monoubiquitination, and that the Tower domain is required for this mechanism. If the FANCD2–FANCI complex is recruited to chromatin in its unmodified form, followed by monoubiquitination ensuring strong retention on chromatin, we should be able to observe a weak recruitment in the absence of ubiquitination. To test this directly, we assessed the recruitment of a mutant of FANCD2 where the lysine that is monoubiquitinated has been mutated, thereby preventing monoubiquitination (FANCD2–K561R). EGFP–FANCD2–K561R was stably expressed in HeLa cells where endogenous FANCD2 was depleted by CRISPR-Cas9-mediated knockout (Supplementary Fig. 5a). As expected, we observed a rapid and weak recruitment of EGFP–FANCD2–K561R (Supplementary Fig. 5b,c). In the same experiment, we observed no recruitment of EGFP–FANCD2ΔTower. Taken together, these data reinforce a model where the FANCD2–FANCI complex is recruited to ICLs via the Tower domain, followed by monoubiquitination while bound to DNA. Of particular interest, we found that three FA patients have disease-causing mutations in the Tower domain^13^, underscoring the functional importance of this domain (Fig. 4e).

### Electrostatic interactions underlie the FANCD2-DNA binding

**Figure 5:**
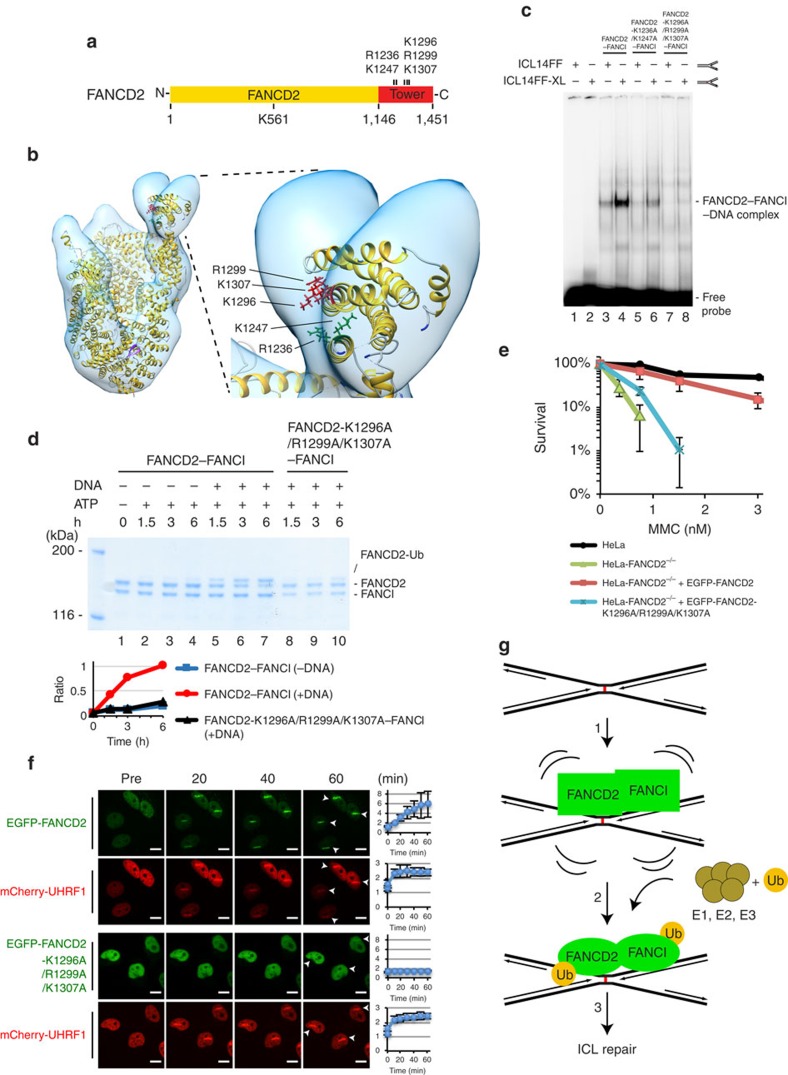
Electrostatic interactions underlie the binding of the FANCD2–FANCI complex to an arrested replication fork at an ICL. (**a**) Schematic of FANCD2 indicating the positive residues, R1236, K1247, K1296, R1299 and K1307, in the Tower domain. (**b**) Zoom-in view of the Tower domain indicating the position of the positive residues. (**c**) EMSA showing that the R1236A and K1247A mutant has weaker DNA binding compared with the wild-type complex, and that the K1296A, R1299A and K1307A mutant has even stronger defect in DNA binding. (**d**) *In vitro* ubiquitination assay of full-length FANCD2–FANCI complex and FANCD2–K1296A/R1299A/K1307A–FANCI complex. Quantification shows the ratio of monoubiquitinated FANCD2 or FANCD2–K1296A/R1299A/K1307A versus unmodified FANCD2 or FANCD2-K1296A/R1299A/K1307A at the indicated time points. (**e**) Clonogenic survival assay of HeLa-FANCD2^−/−^ cells complemented with EGFP–FANCD2 or EGFP–FANCD2–K1296A/R1299A/K1307A. The experiment was done in triplicate. (**f**) HeLa-FANCD2^−/−^ cells expressing EGFP–FANCD2 or EGFP–FANCD2–K1296A/R1299A/K1307A were pre-treated with TMP, and micro-irradiated at the indicated areas (white arrows). mCherry-UHRF1 was co-expressed in the same cells. EGFP–FANCD2 was recruited to ICLs, whereas the recruitment of EGFP–FANCD2–K1296A/R1299A/K1307A was diminished. Representative fields shown. Quantifications of the fluorophore-tagged proteins at the irradiated sites are shown on the right (7 cells quantified for each cell line). Scale bars, 20 μm. (**g**) Model showing how the FANCD2–FANCI complex is recruited to a crosslinked replication fork, triggering the subsequent monoubiquitination of the complex. Error bars in **d**,**e**,**f** show s.d.

To gain further mechanistic insight into how the FANCD2–FANCI complex is recruited to replication forks stalled at ICLs, we carefully analysed the primary amino acid sequence of the Tower domain. Strikingly, we found two patches of positively charged residues that are highly conserved (Supplementary Fig. 6a,b). Inspection of the position of these residues in the FANCD2–FANCI complex showed that they are located on the surface of the Tower domain (Fig. 5a,b). Therefore, we hypothesized that these residues might be defining the interaction of the FANCD2–FANCI complex with the stalled replication fork through electrostatic interactions. To test this directly, we created two mutant versions of FANCD2, one where R1236 and K1247 are mutated and one where K1296, R1299 and K1307 are mutated, all to neutral alanines. We purified recombinant protein complexes of these proteins with FANCI (Supplementary Fig. 6c) and assessed their ability to interact with the ICL-containing DNA structure mimicking a replication fork. As predicted, we observed a significantly reduced binding of the mutant containing two mutated residues, and a nearly complete abrogation of binding when all three residues were mutated (Fig. 5c). When we subjected one of the mutants to the *in vitro* ubiquitination assay, we observed a striking reduction of monoubiquitination, down to the basal level observed in the absence of DNA (Fig. 5d, compare lanes 4, 7 and 10). Since we observed such strong phenotypes *in vitro*, we decided to verify the results *in vivo*. We first assessed the ability of the mutant protein to complement FANCD2^−/−^ HeLa cells. As expected, FANCD2 containing the point mutations abrogating DNA binding and ubiquitination *in vitro*, failed to complement the cellular sensitivity (Fig. 5e). Similarly, when we assessed the ability of this protein to be recruited to ICLs, a clear defect was observed (Fig. 5f). Finally, when we assayed the monoubiquitination status of the mutant version of FANCD2 after introduction of ICLs in live cells, a complete absence of monoubiquitination was observed (Supplementary Fig. 6d).

## Discussion

Activation of FANCD2 by monoubiquitination is an initial and critical event in the FA DNA-repair pathway. The mechanism underlying this process has remained elusive. Here, we present the cryo-EM structure of the full-length human FANCD2–FANCI complex, and uncover the existence of a novel Tower domain of FANCD2. We demonstrate that the Tower domain is required for a direct interaction with a DNA structure mimicking a replication fork arrested at an ICL, and that this interaction triggers the monoubiquitination event. Importantly, our work determines that the FANCD2–FANCI complex is recruited to DNA before it is monoubiquitinated, rather than what was previously thought, namely that the monoubiquitination precedes, and results in, recruitment of the complex to DNA^14^.

The cryo-EM structure of the human FANCD2–FANCI complex is largely in agreement with the previously reported crystal structure of the mouse homologues^8^. The only significant difference is the position of the Tower domain, which appears vertical in our structure, and horizontal in the crystal structure. There are several possible explanations for the difference. First, it might be that the mouse and human proteins fold differently. Second, three deletions were introduced in the mouse FANCD2 protein used for crystal formation, including a deletion of 59 residues at the base of the Tower. It is possible that this region serves as a hinge, which upon deletion causes the Tower to adapt a new confirmation. Third, the FANCD2–FANCI complex used in our structure determination was formed *in vivo* and purified as a complex, whereas the mouse proteins were purified as monomers and the complex assembled *in vitro.* Fourth, the techniques used, hence the state of the protein samples used to obtain the two structures, are principally different, which might affect the conformation that the proteins adapt while being analysed.

It is known that the FANCD2–FANCI complex possesses some DNA-binding activity^8^,^11^,^15^; however, the true substrate during ICL repair has remained elusive. Using purified components we have discovered that the FANCD2–FANCI complex preferentially binds to a DNA structure mimicking a replication fork stalled at an ICL. This finding is in good agreement with existing literature showing a function of the complex during replication-dependent repair of ICLs^16^. We corroborated these results by demonstrating a timely recruitment of FANCD2 to ICLs *in vivo*.

Monoubiquitination of FANCD2 is essential to its function in ICL repair. A point mutation of lysine 561 to arginine, leads to a complete loss of function of the protein. However, the functional consequence of this critical monoubiquitination is unknown. One possibility is that the ubiquitination affects the ability of FANCD2 to be recognized by a protein facilitating the recruitment of FANCD2 to DNA. If that were the case, it would entail that FANCD2 is ubiquitinated before it is recruited to DNA, which is indeed the current understanding. However, we found that monoubiquitination of FANCD2 is strongly stimulated by DNA, suggesting that the protein is first recruited to DNA, likely through binding to a replication fork stalled at an ICL, and thereafter monoubiquitinated (Fig. 5g). Indeed, it has been shown that the FA core complex, that is, the E3 ligase responsible for monoubiquitinating FANCD2, is recruited to chromatin via FANCM and FAAP24 (refs 17, 18), consistent with the notion that ubiquitination takes place on DNA^18^,^19^. It is possible that the FANCD2–FANCI complex undergoes a conformational change upon binding to the ICL, and that this allows the monoubiquitination to take place. Ongoing structural studies should clarify this. It is possible that after FANCD2–FANCI is monoubiquitinated it acquires even higher affinity for the ICL. It is also plausible that the monoubiquitinated form of FANCD2–FANCI interacts with a chromatin-associated factor, ensuring retention after its modification.

## Methods

### Cell lines and antibodies

HeLa (originally from ATCC) and PD20 (kindly provided by the FARF repository) cells were grown in DMEM (D5796, Sigma) supplemented with 2.5–10% fetal bovine serum. Antibodies used were as follows: anti-FANCD2, 1:100 dilution (sc-20022, Santa Cruz Biotechnology); anti-FANCI, 1:1,000 dilution (FARF); anti-FANCA, 1:1,000 dilution (FARF); anti-Lamin B, 1:1,000 dilution (sc-6216, Santa Cruz Biotechnology); anti-Flag, 1:1,000 dilution (M5, F4042, Sigma-Aldrich); anti-Ubiquitin, 1:400 dilution (FK2,BML-PW8810, Enzo Life Sciences); anti-UHRF1, 1:1,000 dilution (sc-373750, Santa Cruz Biotechnology); and anti-a-Tubulin, 1:2,000 dilution (5829, Millipore).

### Plasmids and transfection

EGFP-fused FANCD2 cDNA was expressed using the pOZ-N plasmid^10^. shRNA-mediated knockdown of the FANCD2 gene was achieved by expressing the target sequence 5′-GAGCAAAGCCACTGAGGTA-3′ in the pSuper.retro vector (Clontech). Transfections of plasmid DNA were carried out using FuGENE6 (Promega) according to the manufacturer s instructions. The FANCD2ΔTower mutant protein was generated by deleting amino acids 1,147–1,451 from the full-length protein. Uncropped images of the original Western blots are shown in Supplementary Fig. 7.

### CRISPR-Cas9 gene editing

The targeting sequence used in the sgRNA was: 5′-GTTTGTCTTGTGAGCGTCTGC-3′. HeLa-FANCD2^−/−^ cells were generated using plasmid pX459 (Addgene #48139) as follows^20^: primers, 5′-CACCGTTTGTCTTGTGAGCGTCTGC-3′ and 5′-AAACGCAGACGCTCACAAGACAAAC-3′, were annealed and introduced into the pX459 plasmid through its BbsI site. HeLa cells were transfected with 2 μg of the resulting pX459 plasmid and selected with 4 μg ml^−1^ puromycin after 24 h. After another 24 h, cells were plated at low density and clones were picked after 2 weeks. Clones were analysed using immunoblot analysis.

### Protein purification

FANCD2 and UBA1 were expressed using the pFastBac1 vector (Life Technologies) with an engineered N-terminal Flag-HA tag, and FANCL was expressed using the pFastBac1 vector (Life Technologies) with an engineered N-terminal Flag-MBP tag or a N-terminal Flag-HA tag. For FANCD2–FANCI complex, Sf9 cell pellets were re-suspended in lysis buffer (20 mM Tris-HCl pH 8.0, 0.1 M KCl, 10% glycerol and 0.2 mM PMSF), and sonicated. Lysates were clarified by centrifugation (17,000g), and the supernatants were incubated with M2 anti-FLAG agarose resin (A2220, Sigma) for 2 h. The resin was washed carefully, and the protein was eluted in the same buffer containing 0.5 mg ml^−1^ FLAG peptide. The flag eluate was supplemented with 20 mM Imidazole and incubated with Ni^2+^-NTA (30310, QIAGEN) at 4°C for 2 h with rotation. The resin was washed carefully, and eluted with buffer containing 20 mM Tris (pH 8.0), 0.1 M KCl, 250 mM Imidazole, 0.2 mM PMSF and 10% glycerol. The Ni^2+^-NTA eluate was injected into pre-equilibrated size-exclusion chromatography column, Superdex 200, and eluted with the base buffer containing 20 mM Tris (pH 8.0), 0.1 M KCl and 5% glycerol. The FANCD2ΔTower-FANCI complex is purified as full-length FANCD2–FANCI except the size-exclusion chromatography step. UBA1 and FANCL are purified against FLAG tag described as above. UBE2t expressing plasmid was a kind gift from Dr. Helen Walden, where in brief, UBE2t was cloned into a pET based expression vector containing an N-terminal 6xHis-Smt3 tag. UBE2t was expressed in *E. coli* BL21(DE3) (Novagen). Cells were grown in Lysogeny Broth supplemented with antibiotics at 37 °C. Expression of UBE2t was induced at OD600=0.6 with 0.5mM IPTG, and cultured overnight at 16 °C. Harvested cells were lysed by french press in the buffer containing 0.5 M NaCl, 0.1 M Tris (pH 8), 0.02 M Imidazole, and 0.25 mM TCEP. The lysate was clarified by centrifugation (48,000g), and the supernatant was incubated with Ni-NTA Agarose (QIAGEN) for 2 hours at 4 °C. 6xHis-Smt3 tag was removed overnight at 4 °C by Ulp1 protease at a w/w ratio of 1:15, Ulp1:UBE2t. UBE2t was concentrated, and loaded onto a Superdex 200 column with 200mM NaCl, 0.1M Tris (pH 8) and 10% glycerol. The described UBE2t purification is a derivative of a previously published method^21^.

### EM and 3D image processing

Negatively stained freshly prepared full-length FANCD2–FANCI was applied to glow-discharged, carbon-coated grids and allowed to adsorb for 15–60 s. Specimen was then stained with 2% uranyl acetate. Vitrified full-length FANCD2–FANCI and FANCD2ΔTower–FANCI was prepared on glow-discharged carbon-coated quantifoil grids. Specimen were imaged at a nominal magnification of 30,000 × with a 2 k × 2 k Gatan CCD camera (corresponding to a pixel size of 3.5 Å at the specimen level) in a JEOL 2100, LaB_6_ operating at 200 kV. A preliminary full-length FANCD2–FANCI 3D model was calculated using the RCT^9^ and the WEB and SPIDER software package^22^. For this first 3D model, 4,082 pairs of particles of full-length FANCD2–FANCI were picked from 80 tilt pair images recorded at 50° and 0°. Refinement of the 3D volume obtained from the RCT method was done using images from frozen, hydrated, full-length FANCD2–FANCI. In all, 5,058 particles were selected after using Roseman's algorithm^23^ in SPIDER^22^ procedure, and subsequently manual selection in WEB^22^. Defocus was determined using CTFTILT^24^. 3D classification and refinement were carried out in RELION^25^, with 25 iterations of 1 class and 4 classes 3D classification. For the FANCD2ΔTower–FANCI complex, frozen particles were selected using Roseman's algorithm^23^ in a SPIDER^22^ procedure, with the previous frozen, hydrated, full-length FANCD2–FANCI structure, and then manually screened using the EMAN^26^ program Boxer. In all, 8,547 particles were selected from 71 micrographs with a defocus range of 3–5.2 μm. Defocus was determined using CTFFIND3 (ref. 24). Refinement was carried out using EMAN^26^ software package. The frozen, hydrated, full-length FANCD2–FANCI structure was used as the starting model and refined against the FANCD2ΔTower–FANCI particles. At each iteration, particles with bad cross-correlation values were temporally removed, and intermediate volume was band-pass filtered between 10 and 150 Å. For the resolution limit estimation of 3D-reconstruction volumes, two independent reconstructions were carried out and compared in reciprocal space using increasing shells with the FSC (Fourier shell correlation) technique. Visualization of 3D-density map was done in UCSF Chimera^27^.

### Preparation of interstrand crosslinked DNA substrates

The DNA oligos were annealed in a buffer containing 10 mM Tris-HCl pH 7.5, 100 mM NaCl and 1 mM EDTA. 4,5′,8-trimethylpsoralen (TMP, Sigma, T6137) crosslinking was carried out using 2.6 μM annealed DNA in crosslinking buffer (10 mM Tris-HCl pH 7.5, 1 mM EDTA, and 50 mM NaCl) with 87.6 μM TMP. The reaction was done using a UVA source (4.2 mW m^−2^) wavelength of 365 nm for 6 cycles at 15-min intervals. Our protocol is a modification of a previously published method^28^. ICL was confirmed by 8 M urea 12% denaturing polyacrylmide gel electrophoresis. ICL14FF was created by annealing the following DNA oligonucleotides: ICL14 (+): 5′-CATTGTGAATTCGCCTCTCTGTC**TA**GCCGAAGCTCGAAACGATCTTGTGC-3′; ICL14 (−): 5′-GTCCATCAAAGTTCGACTGTGCGGC**TA**GACAGAGAGGCGAATTCACAATG-3′, ICL14 leading: 5′-GCACAAGATCGTTTCGAGCTT-3′, ICL14 lagging: 5′-GTCGAACTTTGATGGAC-3′. The bases part of the ICL are indicated in bold.

### Electrophoretic mobility shift assay

The protein–DNA-binding reaction that contained 0.5 μg of FANCD2–FANCI complex, or derivative thereof, and 1 nM radiolabeled DNA, was performed in 10 μl containing 25 mM Tris-HCl pH 7.5, 100 mM NaCl, 6% glycerol and 1 mM dithiothreitol. The reaction mixture was incubated at room temperature for 1 h. For super-shift, 0.5 μg anti-HA antibody was added, and incubated for another 1 h. For the experiment in Fig. 4c the following amounts of protein were used: 0, 0.125, 0.25 and 0.5 μg. After the incubation, the reaction mixtures were loaded onto a 0.4 × TBE, 4% polyacrylamide gel, and run with 0.4 × TBE buffer at 4 °C. The gel was then fixed with 10% methanol/10% acetic acid for 5 min, dried and exposed to film or photostimulable phosphor imaging plate.

### Live-cell imaging

EGFP-fused wild-type and mutant FANCD2 or mCherry-fused UHRF1 cDNA were inserted into the pOZ vector as described above. Live-cell imaging were carried out with an OLYMPUS IX81 microscope connected to PerkinElmer UltraView Vox spinning-disk system equipped with a Plan-Apochromat 60 × /1.4 oil objective using Volocity software 6.3 for image capturing. EGFP was excited with 488-nm laser lines. Throughout the experiment, these cells were maintained at 5% CO_2_, and 37 °C using a live-cell environmental chamber (Tokai hit). Confocal image series were typically recorded with a frame size of 512 × 512 pixels and a pixel size of 139 nm. For localized DNA-damage induction, cells were seeded in glass-bottom dish (MatTek) and sensitized by incubation in DMEM supplemented with 10% fetal bovine serum and 20** **μg ml^−1^ TMP for 30 min at 37 °C. Microirradiation was performed using the FRAP preview mode of the Volocity software by scanning (each irradiation time was 100 ms) a pre-selected 3–5 stripes (50 × 3 pixels) within the nucleus 10–60 times with a 405-nm laser set to 100% laser power. The EGFP and mCherry intensities at micro-irradiated sites were quantified using ImageJ with Fiji, and normalized by their intensities before microirradiation.

### Cell fractionation and whole-cell lysate

Collected cell pellets were permeabilized with CSK buffer containing 200 mM NaCl, 10 mM PIPES, 300 mM Sucrose, 1 mM MgCl_2_, 1 mM EDTA and 0.5% Triton X-100 on ice for 10 min. CSK fraction (supernatant) and nuclear pellet were separated by centrifugation at 900*g* at 4°C for 10 min. Nuclear pellet was processed the same way as whole-cell lysate described below. Preparation of whole-cell lysate was performed by scraping off cells from dishes followed by centrifugation at 100*g* for 5 min. Cell pellets were re-suspended and incubated in an equal volume of Benzonase buffer (2 mM MgCl_2_, 20 mM Tris-HCl (pH 8.0), 10% glycerol, 1% Triton X-100 and 12.5 U ml^−1^ Benzonase (E1014, Sigma-Aldrich) on ice for 10 min. The cells were then lysed by addition of an equal volume of 2% SDS to reach a final concentration of 1%. Samples were heated at 70 °C for 2 min. The protein concentration was determined by the Bradford assay (Bio-Rad Life Science).

### Clonogenic survival assay

Cells (250–4,000) were plated in six-well plates and treated with different dosages of MMC on the next day. Colony formation was scored after 10–14 days using 1% (w/v) crystal violet in methanol.

### Co-immunoprecipitation

PD20 and PD20-expressing FLAG-HA-tagged FANCD2 and FANCD2ΔTower cells were treated with 160 ng ml^−1^ MMC overnight. Cell pellets were incubated with Buffer A (0.5% Triton X-100, 20 mM Tris pH 8.0, 2 mM MgCl_2_, 5 mM MgCl_2_, 10% Glycerol, 50 U μl^−1^ Benzonase for 10 min on ice. Ten times the pellet volume of Buffer B (0.5% Triton X-100, 20 mM Tris-HCl pH 8.0, 2 mM MgCl_2_, 10% Glycerol, 150 mM KCl and 0.2 mM PMSF) was added to the mixture and incubated for 10 min for extraction. Lysates were clarified by centrifugation (17,000g), and supernatant was used for immunoprecipitation. M2 agarose beads were added to the lysates, and incubated for 2 h. The resin was washed extensively, and eluted with 0.5 mg ml^−1^ FLAG peptide.

### *In vitro* ubiquitination assay

Reaction volumes of 25 μl contained 17 nM UBA1, 0.64 mM UBE2T, 0.372 mM FANCL or FLAG-HA-FANCL, 4.2 mM His-Ub, 0.25 mM FANCD2–FANCI complex or derivatives thereof, 20 mM pBlueScript SKII (+) when indicated, in the following reaction buffer: 50 mM Tris (pH 7.5), 100 mM KCl_2_, 2 mM MgCl_2_, 0.5 mM dithiothreitol and 2 mM ATP. Reactions were incubated at room temperature for the indicated time. In all, 5 μl of 6 × SDS loading buffer containing BME was used to terminate reactions. Samples were loaded onto an SDS-PAGE gel and subjected to Coomassie blue staining or immunoblotting. The described *in vitro* ubiquitination assay is a derivative of a previously published method^21^.

### Data availability

Cryo-EM reconstructions of the FANCD2–FANCI and FANCD2ΔTower–FANCI complexes are deposited to the EMDB database under accession numbers EMD-8141 and EMD-8142, respectively.

## Additional information

**How to cite this article:** Liang, C.-C. *et al.* The FANCD2–FANCI complex is recruited to DNA interstrand crosslinks before monoubiquitination of FANCD2. *Nat. Commun.* 7:12124 doi: 10.1038/ncomms12124 (2016).

## Supplementary Material

Supplementary Information Supplementary Figures 1-7

Peer Review File

## References

[b1] Jo U. & Kim H. Exploiting the Fanconi anemia pathway for targeted anti-cancer therapy. Mol. Cells. 38, 669–676 (2015). 26194820 10.14348/molcells.2015.0175 PMC4546938

[b2] Garcia-Higuera I. *et al.* Interaction of the Fanconi anemia proteins and BRCA1 in a common pathway. Mol. Cell. 7, 249–262 (2001). 11239454 10.1016/s1097-2765(01)00173-3

[b3] Kratz K. *et al.* Deficiency of FANCD2-associated nuclease KIAA1018/FAN1 sensitizes cells to interstrand crosslinking agents. Cell 142, 77–88 (2010). 20603016 10.1016/j.cell.2010.06.022

[b4] MacKay C. *et al.* Identification of KIAA1018/FAN1, a DNA repair nuclease recruited to DNA damage by monoubiquitinated FANCD2. Cell 142, 65–76 (2010). 20603015 10.1016/j.cell.2010.06.021 PMC3710700

[b5] Smogorzewska A. *et al.* A genetic screen identifies FAN1, a Fanconi anemia-associated nuclease necessary for DNA interstrand crosslink repair. Mol. Cell. 39, 36–47 (2010). 20603073 10.1016/j.molcel.2010.06.023 PMC2919743

[b6] Hodskinson M. R. *et al.* Mouse SLX4 is a tumor suppressor that stimulates the activity of the nuclease XPF-ERCC1 in DNA crosslink repair. Mol. Cell. 54, 472–484 (2014). 24726326 10.1016/j.molcel.2014.03.014 PMC4017094

[b7] Klein Douwel D. *et al.* XPF-ERCC1 acts in unhooking DNA interstrand crosslinks in cooperation with FANCD2 and FANCP/SLX4. Mol. Cell. 54, 460–471 (2014). 24726325 10.1016/j.molcel.2014.03.015 PMC5067070

[b8] Joo W. *et al.* Structure of the FANCI-FANCD2 complex: insights into the Fanconi anemia DNA repair pathway. Science 333, 312–316 (2011). 21764741 10.1126/science.1205805 PMC3310437

[b9] Radermacher M., Wagenknecht T., Verschoor A. & Frank J. Three-dimensional reconstruction from a single-exposure, random conical tilt series applied to the 50S ribosomal subunit of *Escherichia coli*. J. Microsc. 146, 113–136 (1987). 3302267 10.1111/j.1365-2818.1987.tb01333.x

[b10] Liang C. C. *et al.* UHRF1 is a sensor for DNA interstrand crosslinks and recruits FANCD2 to initiate the Fanconi anemia pathway. Cell Rep. 10, 1947–1956 (2015). 25801034 10.1016/j.celrep.2015.02.053 PMC4386029

[b11] Sato K., Toda K., Ishiai M., Takata M. & Kurumizaka H. DNA robustly stimulates FANCD2 monoubiquitylation in the complex with FANCI. Nucleic Acids Res. 40, 4553–4561 (2012). 22287633 10.1093/nar/gks053 PMC3378891

[b12] Longerich S. *et al.* Regulation of FANCD2 and FANCI monoubiquitination by their interaction and by DNA. Nucleic Acids Res. 42, 5657–5670 (2014). 24623813 10.1093/nar/gku198 PMC4027212

[b13] Kalb R. *et al.* Hypomorphic mutations in the gene encoding a key Fanconi anemia protein, FANCD2, sustain a significant group of FA-D2 patients with severe phenotype. Am. J. Hum. Genet. 80, 895–910 (2007). 17436244 10.1086/517616 PMC1852747

[b14] Kottemann M. C. & Smogorzewska A. Fanconi anaemia and the repair of Watson and Crick DNA crosslinks. Nature 493, 356–363 (2013). 23325218 10.1038/nature11863 PMC3700363

[b15] Park W. H. *et al.* Direct DNA binding activity of the Fanconi anemia D2 protein. J. Biol. Chem. 280, 23593–23598 (2005). 15849361 10.1074/jbc.M503730200

[b16] Knipscheer P. *et al.* The Fanconi anemia pathway promotes replication-dependent DNA interstrand cross-link repair. Science 326, 1698–1701 (2009). 19965384 10.1126/science.1182372 PMC2909596

[b17] Ciccia A. *et al.* Identification of FAAP24, a Fanconi anemia core complex protein that interacts with FANCM. Mol. Cell. 25, 331–343 (2007). 17289582 10.1016/j.molcel.2007.01.003

[b18] Kim J. M., Kee Y., Gurtan A. & D'Andrea A. D. Cell cycle-dependent chromatin loading of the Fanconi anemia core complex by FANCM/FAAP24. Blood 111, 5215–5222 (2008). 18174376 10.1182/blood-2007-09-113092 PMC2384144

[b19] Castella M. *et al.* FANCI regulates recruitment of the FA core complex at sites of DNA damage independently of FANCD2. PLoS. Genet. 11, e1005563 (2015). 26430909 10.1371/journal.pgen.1005563 PMC4592014

[b20] Ran F. A. *et al.* Genome engineering using the CRISPR-Cas9 system. Nat. Protoc. 8, 2281–2308 (2013). 24157548 10.1038/nprot.2013.143 PMC3969860

[b21] Hodson C., Purkiss A., Miles J. A. & Walden H. Structure of the human FANCL RING-Ube2T complex reveals determinants of cognate E3-E2 selection. Structure 22, 337–344 (2014). 24389026 10.1016/j.str.2013.12.004 PMC3979106

[b22] Frank J. *et al.* SPIDER and WEB: processing and visualization of images in 3D electron microscopy and related fields. J. Struct. Biol. 116, 190–199 (1996). 8742743 10.1006/jsbi.1996.0030

[b23] Roseman A. M. Particle finding in electron micrographs using a fast local correlation algorithm. Ultramicroscopy 94, 225–236 (2003). 12524193 10.1016/s0304-3991(02)00333-9

[b24] Mindell J. A. & Grigorieff N. Accurate determination of local defocus and specimen tilt in electron microscopy. J. Struct. Biol. 142, 334–347 (2003). 12781660 10.1016/s1047-8477(03)00069-8

[b25] Scheres S. H. RELION: implementation of a Bayesian approach to cryo-EM structure determination. J. Struct. Biol. 180, 519–530 (2012). 23000701 10.1016/j.jsb.2012.09.006 PMC3690530

[b26] Ludtke S. J., Baldwin P. R. & Chiu W. EMAN: semiautomated software for high-resolution single-particle reconstructions. J. Struct. Biol. 128, 82–97 (1999). 10600563 10.1006/jsbi.1999.4174

[b27] Pettersen E. F. *et al.* UCSF Chimera–a visualization system for exploratory research and analysis. J. Comput. Chem. 25, 1605–1612 (2004). 15264254 10.1002/jcc.20084

[b28] Esposito F., Brankamp R. G. & Sinden R. R. DNA sequence specificity of 4,5',8-trimethylpsoralen cross-linking. Effect of neighboring bases on cross-linking the 5'-TA dinucleotide. J. Biol. Chem. 263, 11466–11472 (1988). 2841329

