# Supplementary material for: The FANCD2–FANCI complex is recruited to DNA interstrand crosslinks before monoubiquitination of FANCD2
Source: Nat Commun. 2016 Jul 13;7:12124. doi: 10.1038/ncomms12124 (PMC4947157; doi:10.1038/ncomms12124)
Supplement: Supplementary Information — Supplementary Figures 1-7 [file ncomms12124-s1.pdf]

Supplementary Figure 1

a b

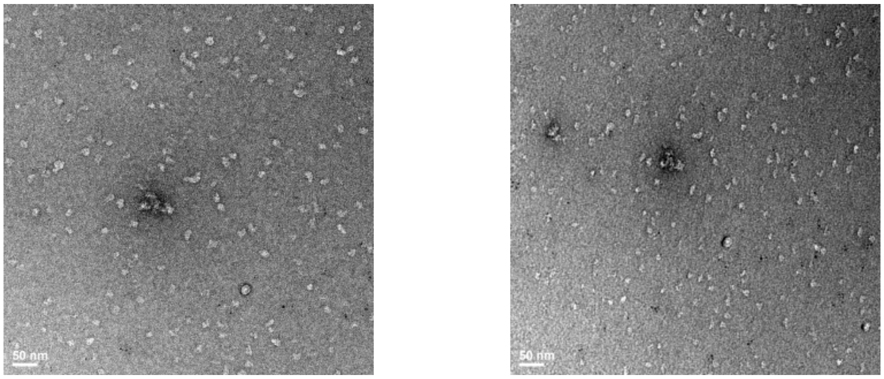

Supplementary Figure 1. Representative micrographs showed homogeneity of the sample. A) Micrograph of negative staining EM full-length FANCD2-FANCI complex 0° tilt. B) Tilted micrograph (50° tilt) of the same area as in (A).

Supplementary Figure 2

a

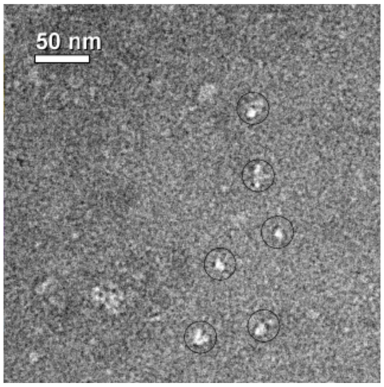

b

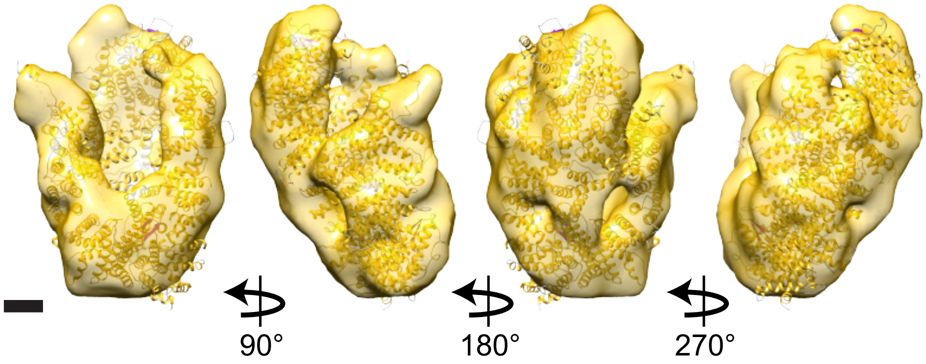

Supplementary Figure 2. Comparison of the human FANCD2-ΔTower-FANCI complex and the mouse complex. A) Micrograph of Cryo-EM of full-length FANCD2-FANCI complex. B) Different orientations of Cryo-EM density map of FANCD2-ΔTower-FANCI complex docked with the mouse FANCD2-FANCI crystal structure deleting amino acids 1144 – 1450 (PDB: 3S4W). Scale bar, 20 Å.

Supplementary Figure 3

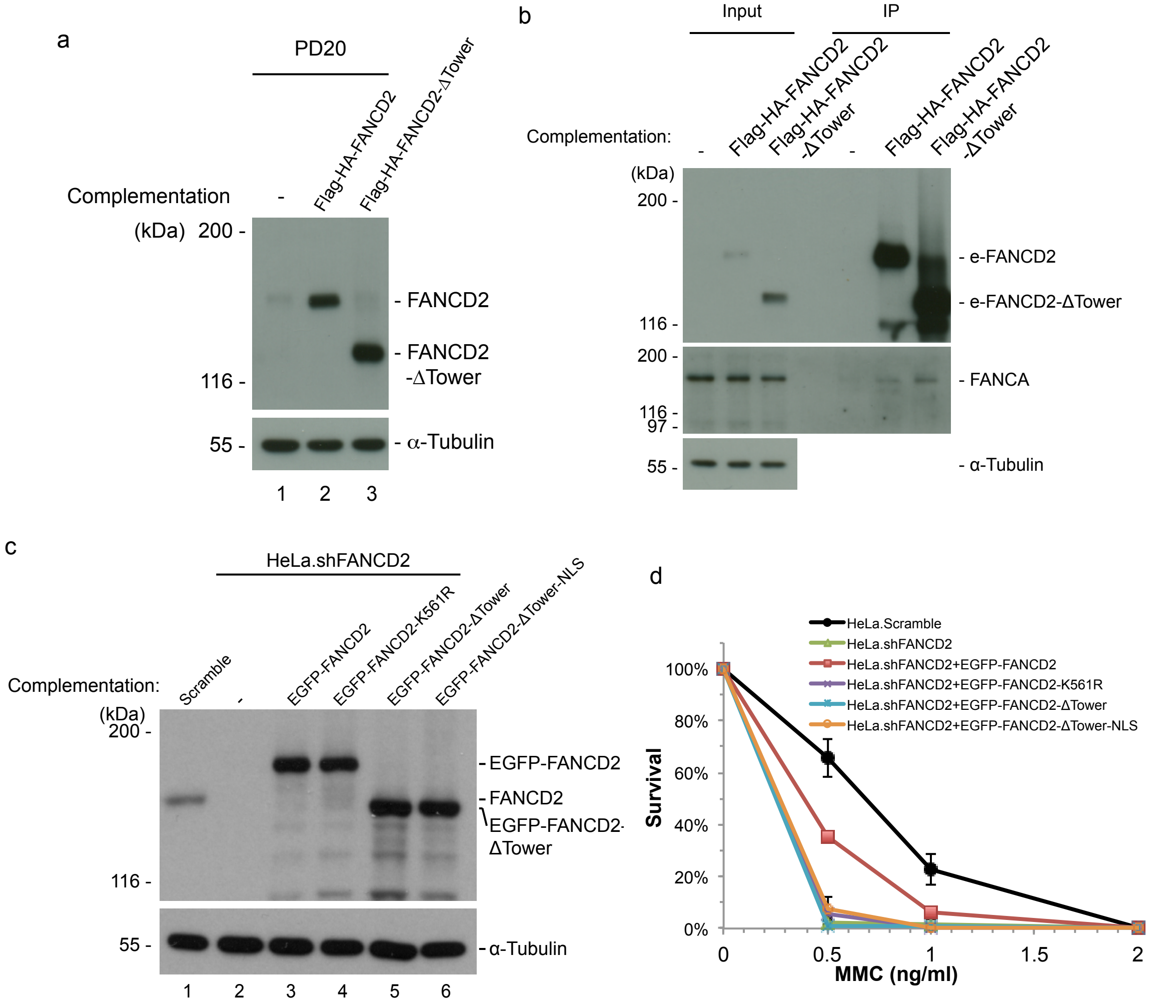

Supplementary Figure 3. The Tower domain of FANCD2 is functionally important but dispensable for the interaction with the FA core complex. A) Western blot analysis of PD20 cells complemented with Flag-HA-FANCD2 and Flag-HA-FANCD2-ΔTower used in Figure 3A showing the expression level of FANCD2. B) Immunoprecipitation of Flag-HA-FANCD2 and Flag-HA-FANCD2-ΔTower from PD20 cells, immunoblotting against FANCD2 and FANCA (component of the FA core complex). PD20 without complementation was used as a negative control. Cells were treated overnight with 160ng/ml MMC. C) Western blotting of the HeLa.Scramble, HeLa.shFANCD2 and HeLa.shFANCD2 complemented with EGFP tagged wild type, K561R, ΔTower and ΔTower-NLS FANCD2 used in Figure S3D showing the expression level of FANCD2. D) Clonogenic survival assay of HeLa.Scramble, HeLa.shFANCD2 and HeLa.shFANCD2 complemented with EGFP tagged wild type, K561R, ΔTower and ΔTower-NLS FANCD2. The experiment was done in triplicate. Error bars show SD.

Supplementary Figure 4

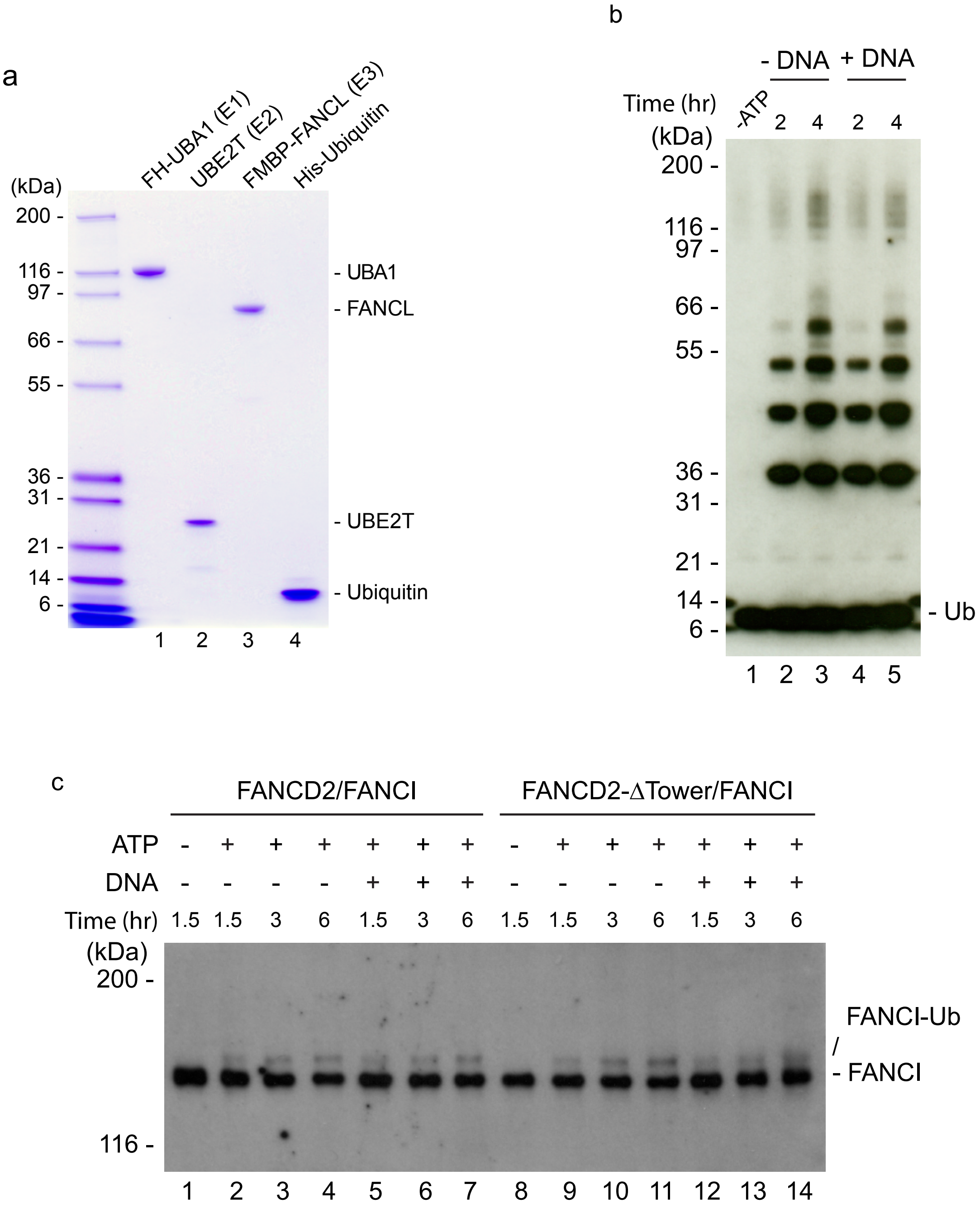

Supplementary Figure 4. The Tower domain of FANCD2 is important for monoubiquitination of FANCD2. A) Coomassie blue stain of purified recombinant FLAG-HA-UBA1 (E1), UBE2T (E2), FLAG-MBP-FANCL (E3) and 6xHis-ubiquitin. B) *In vitro* ubiquitination assay of full-length FANCD2-FANCI complex. Immunoblot using antibodies against Ubiquitin. C) *In vitro* ubiquitination assay of full-length FANCD2-FANCI complex. Immunoblot using antibodies against FANCI.

Supplementary Figure 5

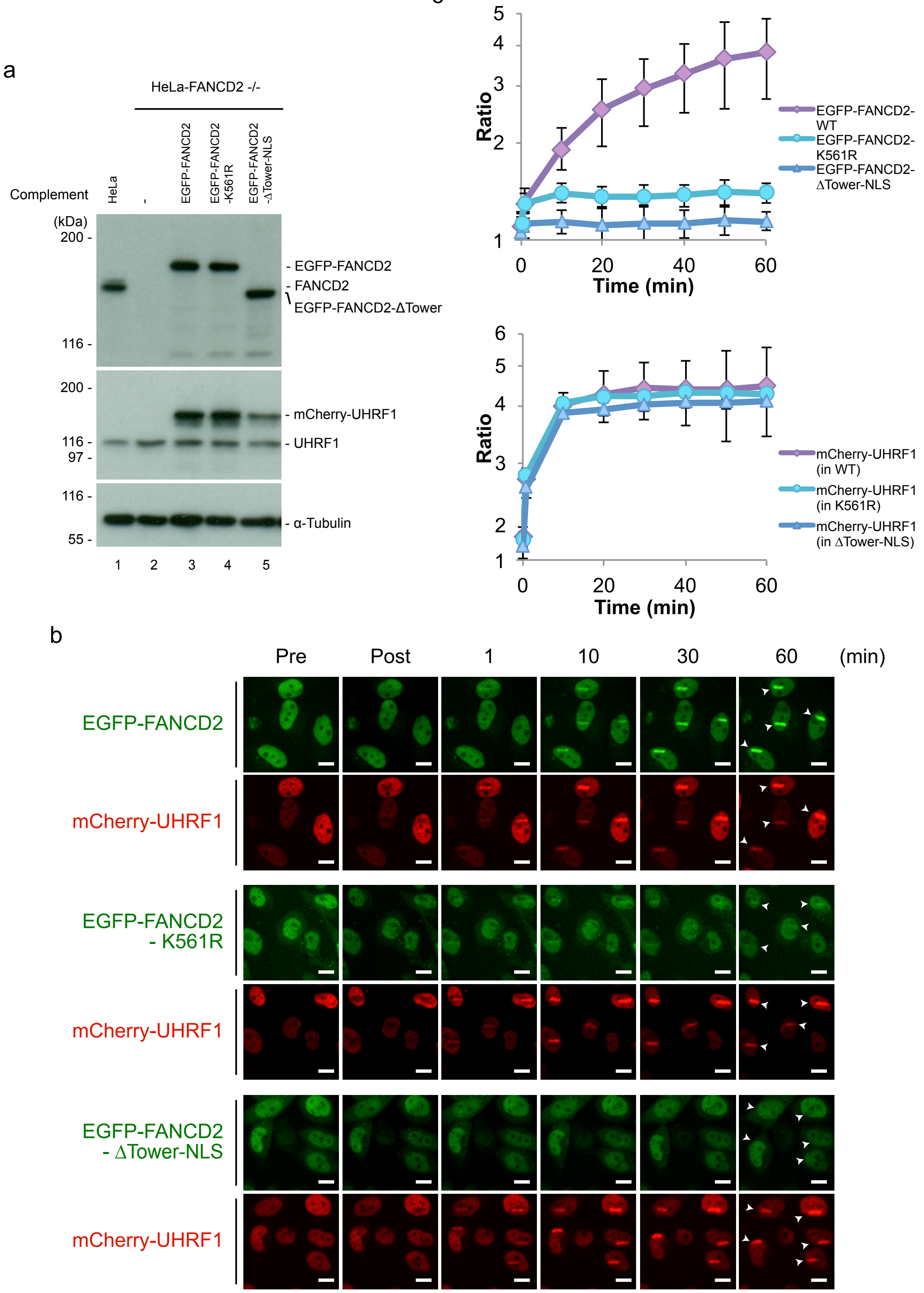

Supplementary Figure 5. FANCD2 recruitment to DNA through the Tower domain precedes its monoubiquitination on lysine 561. A) Western blot analysis of HeLa-FANCD2 -/- complemented with mCherry-UHRF1 and EGFP-tagged wild type, K561R and ΔTower-NLS FANCD2 used in Figure S5B showing the expression level of UHRF1 and FANCD2. B) HeLa-FANCD2 -/- cells expressing EGFP-tagged wild type, K561R and ΔTower-NLS FANCD2 were pre-treated with TMP, and micro-irradiated at the indicated areas (white arrows). mCherry-UHRF1 was co-expressed in all three cell lines and used as positive controls for the introduction of ICLs. Wild type EGFP-FANCD2 was recruited to ICLs rapidly and strongly, EGFP-FANCD2-K561R was recruited rapidly and weakly, while no recruitment of EGFP-FANCD2-ΔTower-NLS was observed. Representative fields shown. C) Quantification of the fluorophore-tagged proteins recruited to ICLs at the irradiated sites (8, 11 and 10 cells were quantified for EGFP-FANCD2, EGFP-FANCD2-K561R and EGFP-FANCD2-ΔTower-NLS, respectively). Scale bar: 20μm. Error bars show SD.

Supplementary Figure 6

a

R1236 K1247

```
gi|Homo         LINSPK-DASSSTFPTLTRHTFVVFFRVMMAELEKTVKKIEPGT-AADSQQIHEEKLLYW  1268
gi|Pan          LINSPK-DASSSTFPTLTRHTFVVFFRVMMAELEKTVKKIEPGT-AADSQQIHEEKLLYW  1286
gi|Macaca       LINSPK-DVSSSTFPTLTRHTFVVFFRVMMAELEKTVKKIEPGT-AADSQQIHEEKLLYW  1198
gi|Sus          LINSPTKDASSSTFPTLTRHTFVIFFRVMMAELEKTVKGLQVAT-AADSQQIHEEKLLYW  1268
gi|Loxodonta    LINSPK-DASSSTFPTLTRHTFVIFFRVMMAELEKTVKGLQAGT-ATDQQQVHEEKLLYW  1268
gi|Mus          LVSAPK-DAASSTFPTLTRHTFVIFFRVMMAELEKTVKGLQAGT-AADSQQVHEEKLLYW  1266
gi|Monodelphis  LIQSSK-DASSSSFPTLTRQTFVVFFRVMMAELEKSVRSLQAGT-AADLPKVHEEKLLYW  1270
gi|Rattus       LVNAPK-DASSSTFPTLTRHTFVIFFRVMMAELEKTVKGLQAGT-ATDSQQVHEEKLLYW  1271
gi|Bos          LINSPK-EASSSTFPTLTWTTFVIFFRVMMAELEKTVKGLQAGT-AADSQQIHEEKLLYW  1282
gi|Equus        LINSPK-DASSSTFPTLTRHTFVIFFRVMMAELEKTVKGLQAGT-AADSQQIHEEKLLYW  1266
gi|Canis        LINSPK-DASSSMFPTLTRQSFVIFFRVMMAELEKTVKGLQAGT-AADSQQIHEEKLLYW  1265
gi|Ailuropoda   LINSPK-DASSSTFPTLTRHTFVIFFRVMMAELEKTVKGLQAGT-AADAQQIHEEKLLYW  1268
gi|Meleagris    LINCAK-DGCSSTYPTLSRQTFPVFFRVMMAQLESSVKSIPAGK-PSDSSEVQLEKLLKW  1265
gi|Gallus       LINSAK-DGCSSTYPTLSRQTFPVFFRVMMAQLESSVKSIPAGK-PSDSGEVQLEKLLKW  1268
gi|Drosophila   ILNT-K-DKALTSFPNFKKANFPLLFRGLCEVLIHSLSGQVS-------VDSRGDKLKLW  1287
gi|Bombyx       LLES-K-NDTLRGLKCINKVNFPIIFRNLGTAVFE------AAKRRLDKGMTNAEHLVLW  1165
gi|Anolis       LINSSK-DGYSSTYPTLTRQTFPIFFRVMMTQLESSIKSIPSGTASSDTREVQLEKLLQW  1312
gi|Xenopus      LVNAAK-DAASSSYPTLTRQTFVVFFRVMMDKLEKCVKSIPNSK-KAETLQEQTEQLLSW  1261
gi|Danio        LLNAAK-DANSRSWPTLNRQTFLVYYKCMMSELEKAVRKIPPSK-QMDNQEMQSEKLLTW  1270
                ::.  . :        :.  .* : :: :    :                  . ::*  *
```

b

K1296 K1299 K1307

```
gi|Homo         NMAVRDFSILINLIKVFDSHPVLHVCLKYGRLFVEAFLKQCMPLLDFSFRKHREDVLSLL  1328
gi|Pan          NMAVRDFSILINLIKVFDSHPVLHVCLKYGRLFVEAFLKQCMPLLDFSFRKHREDVLSLL  1346
gi|Macaca       NMAVRDFSILINLIKVFDSHPVLHVCLKYGRLFVEAFLKQCMPLLDFSFRKHREDVLSLL  1258
gi|Sus          NMAVRDFSILINLIKVFDSRPVLHICLKYGRLFVEAFLKQCMPLLDFSFRKHREDVLSLL  1328
gi|Loxodonta    NMAVRDFSILLNLIKVFDSHPVLHVCLKYGRLFVEAFLKQCMPLLDFSFRKHREDVLSLL  1328
gi|Mus          NMAVRDFSILLNLMKVFDSYPVLHVCLKYGRRFVEAFLKQCMPLLDFSFRKHREDVLSLL  1326
gi|Monodelphis  SMAVRDFNILINLVKVFDSRPVLNVCLKYGRLFVETFLKQCMPLLDFSFRKHREDVLSLL  1330
gi|Rattus       NMAVRDFSILINLMKVFDSYPVLHVCLKYGRRFVEAFLKQCMPLLDFSFRKHRDDVLSLL  1331
gi|Bos          NMAVRDFSILINLIKVFDSRPVLFACLKYGRLFVEAFLKQCMPLLDFSFRKHREDVLSLL  1342
gi|Equus        NMAVRDFSILINLIKVFDSRPVLHVCLKYGRLFVEAFLKQCMPLLDFSFRKHREDVLSLL  1326
gi|Canis        NMAVRDFSILINLIKVFDSRPVLHVCLKYGRLFVEAFLKQCMPLLDFSFRKHREDVLSLL  1325
gi|Ailuropoda   NMAVRDFSILINLIKVFDSRPVLHVCLKYGRLFVEAFLKQCMPLLDFSFRKHREDVLSLL  1328
gi|Meleagris    NIAVRNFHILINLVKVFDSRPVLSICLKYGRLFVEAFLKHAMPLLDHSFKKHRDDVQSLL  1325
gi|Gallus       NIAVRNFHILINLVKVFDSRPVLSICLKYGRLFVEAFLKLAMPLLDHSFKKHRDDVQSLL  1328
gi|Drosophila   ESAVDLLNGLLSIVQQVEQPRNFGLFLKHSLLFLKLLLQHGMSALESIVREDPERLTRFL  1347
gi|Bombyx       RDIADILKSMTEIVKTQKSN-SLTAFFKNSMPILRLFTLQGIPILKLEFKNQTDEVLQIL  1224
gi|Anolis       NMAVRDFHILVNLVKVFDSRPVLSVCLKYGRLFVETFLKHGMPLLDYSFKKHREDVQSLL  1372
gi|Xenopus      NLAVRDFHILVNLVKVFDSRPVLSICLKYGRLFVETFLKLGMPLLDCCFKKQREDVQSLL  1321
gi|Danio        NLAVRDFHILINLVKMFDSRPVLTVCLKYGRLFLESFLKLGMPLLDYSFKKHKEDVQGLL  1330
                   .  :  : .:::  ..   :    :* .  ::. :    :  *.  .::. : :  :*
```

c

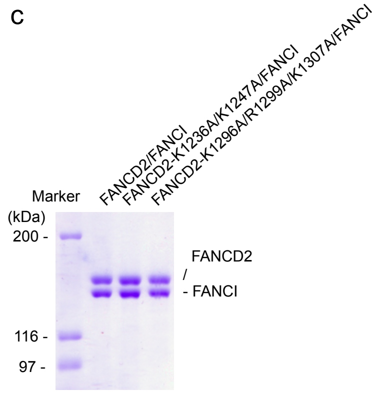

d

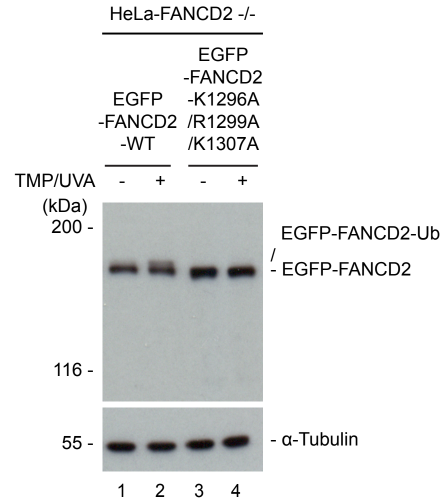

Supplementary Figure 6. Residues K1296, R1299 and K1307 of the Tower domain of FANCD2 are highly conserved and critical for the *in vivo* ubiquitination of FANCD2. A) Sequence alignment of the C-terminal region of FANCD2 showing the conservation of the positive residues, R1236 and K1247 in the Tower domain. B) Sequence alignment of the C-terminal region of FANCD2 showing the conservation of the positive residues, K1296, R1299 and K1307, in the Tower domain. C) Coomassie blue stain of purified recombinant wild type, R1236A/K1247A and K1296A/R1299A/K1307A FANCD2-FANCI complex. D) HeLa-FANCD2 -/- cells complemented with EGFP-FANCD2 and EGFP-FANCD2 K1296A/R1299A/K1307A were treated with 500ng TMP, irradiated with 250J/m^2, 365nm UVA. Cells were harvested after 6 hours followed by whole cell lysis and immunoblotting using the indicated antibodies.

Supplementary Figure 7

Figure 3b

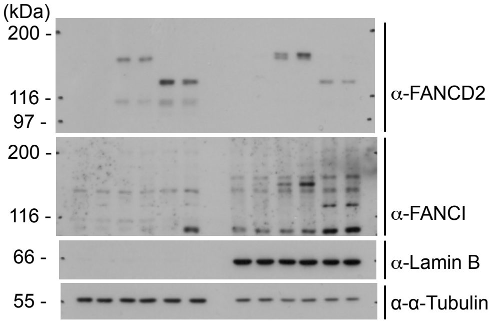

Figure 4d

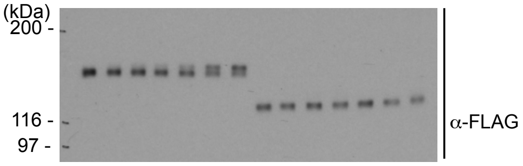

Supplementary Figure 3a

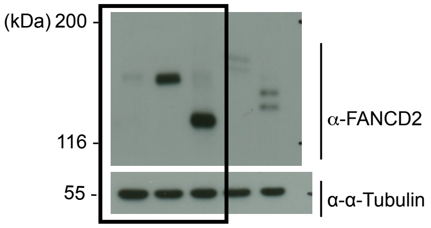

Supplementary Figure 3b

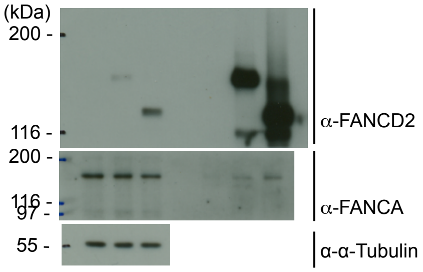

Supplementary Figure 3c

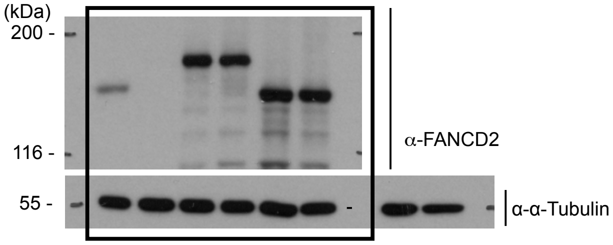

Supplementary Figure 4b

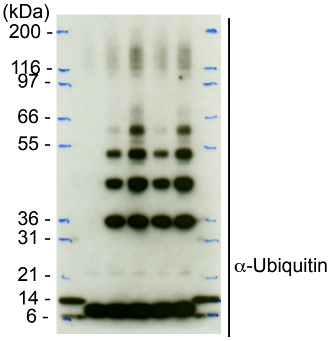

Supplementary Figure 4c

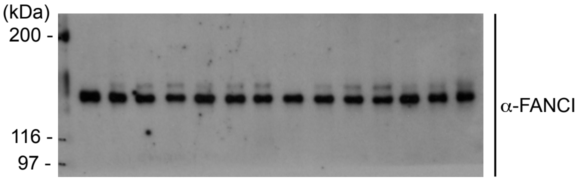

Supplementary Figure 5a

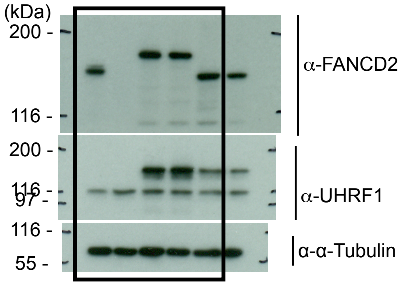

Supplementary Figure 6d

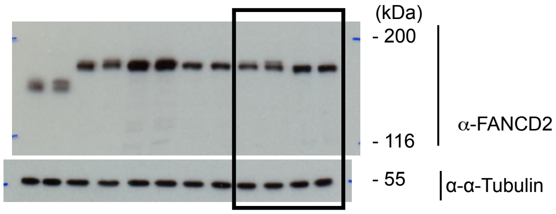

Supplementary Figure 7. Uncropped original images of immunoblotting results.
