# Supplementary material for: The FANCD2–FANCI complex is recruited to DNA interstrand crosslinks before monoubiquitination of FANCD2
Source: Nat Commun. 2016 Jul 13;7:12124. doi: 10.1038/ncomms12124 (PMC4947157; doi:10.1038/ncomms12124)
Supplement: Peer Review File [file ncomms12124-s2.pdf]

Editorial Note: this manuscript has been previously reviewed at another journal that is not operating a transparent peer review scheme. This document only contains reviewer comments and rebuttal letters for versions considered at Nature Communications.
